# Development of a duplex droplet digital PCR assay for absolute quantitative detection of "*Candidatus* Liberibacter asiaticus"

**DOI:** 10.1371/journal.pone.0197184

**Published:** 2018-05-17

**Authors:** Vijayanandraj Selvaraj, Yogita Maheshwari, Subhas Hajeri, Jianchi Chen, Thomas Greg McCollum, Raymond Yokomi

**Affiliations:** 1 USDA-ARS, San Joaquin Valley Agricultural Sciences Center, Parlier, CA, United States of America; 2 Citrus Pest Detection Program, Central California Tristeza Eradication Agency, Tulare, CA, United States of America; 3 USDA-ARS, U.S. Horticultural Research Laboratory, Fort Pierce, FL, United States of America; Oklahoma State University, UNITED STATES

## Abstract

Huanglongbing (HLB, citrus greening) is a devastating citrus disease affecting citrus production worldwide. It is associated with the bacterium “*Candidatus* Liberibacter asiaticus” (*C*Las) and is vectored by the Asian citrus psyllid (ACP). Currently, diagnosis of *C*Las in regulatory samples is based on real-time quantitative polymerase chain reaction (qPCR) using 16S rRNA gene specific primers/probe. The detection of *C*Las using qPCR is challenging due to low pathogen titer and uneven distribution in infected plants and exacerbated by sampling issues and presence of inhibitors. This study evaluated a duplex droplet digital polymerase chain reaction (ddPCR) using multi-copy gene targets, 16S and RNR, to simultaneously detect *C*Las DNA targets in the same sample for unambiguous detection of the HLB pathogen in DNA extracts from citrus leaves and ACP. Standard curve analyses on tenfold dilution series with plasmid, citrus leaf and ACP DNA showed that both ddPCR and qPCR exhibited good linearity and efficiency in the duplex assay. *C*Las-infected low titer samples were used to validate the duplex ddPCR and qPCR performance and demonstrated that detection rate is higher when both 16S and RNR primers were used in duplex assay. However, the receiver operating characteristic analysis indicated that area under the curve for RNR primer was significantly broader, compared to 16S primers for *C*Las detection at low target titer. The absolute quantification of *C*Las at variable titers was reproducible and repeatable for both primer sets and the ddPCR showed higher resilience to PCR inhibitors with citrus leaf and ACP extracts. Hence, the resultant duplex ddPCR assay resulted in a significantly improved detection platform for diagnosis of *C*Las in samples with low pathogen titer.

## Introduction

“*Candidatus* Liberibacter asiaticus” (*C*Las) is a Gram-negative, α –proteobacterium [1] associated with a devastating citrus disease known as Huanglongbing (HLB) (aka citrus greening) which is present worldwide except in the Australia, New Zealand and European Union countries [2]. HLB is characterized by blotchy mottling of young leaves with yellow shoots, malformed fruit, twig dieback and tree decline. This fastidious bacterium resides in phloem sieve cells of host plants. The bacterium causes multiple pockets of necrotic phloem leading to blockage of translocation and accumulation of starch in plastids, aberrations in cambial activity and excessive phloem formations observed in the infected leaf tissues under light microscopy [3]. *C*Las is vectored by the Asian citrus psyllid (ACP) (*Diaphorina citri*).

The detection of *C*Las is challenging due to low titer with uneven distribution *in planta*. HLB symptoms can be confused with nutritional deficiencies or other pathogens such as *Spiroplasma citri*, causal agent of citrus stubborn disease in California and other arid citrus-growing regions. Following *C*Las infection, there is a latency period of several months to multiple years before HLB symptoms appear and trees become unproductive or can die. Currently, no sustainable remedies are available for effective management of HLB. In *C*Las quarantine areas, infected trees are removed at the earliest stage possible to prevent further acquisition and spread of *C*Las by ACP. A robust and accurate detection method plays an important role in reduction of inoculum and limiting disease spread.

The available nucleic acid-based detection methods such as electron microscopy [4], fluorescence imaging techniques [5], and loop mediated isothermal amplification [6, 7] results are not sensitive at low titers. The chromosome of *C*Las has three copies of the 16S rRNA gene [8] and five copies of *nrdB*, encoding the β-subunit of ribonucleotide reductase (RNR), a critical enzyme involving bacterial proliferation [9]. Polymerase chain reaction (PCR), real-time PCR (qPCR) and ddPCR have been developed for 16S rDNA [10, 11, 12]. *C*Las detection using the RNR gene by PCR [13] and qPCR [9] was developed.

Droplet digital PCR (ddPCR) has been widely used in medical and clinical research in recent years for absolute quantification of nucleic acids without dependence on external standard curves [14]. The PCR reaction containing DNA templates, primers and a fluorescently labeled hydrolysis probe or a nucleic acid intercalating dye (Eva-Green) is partitioned into thousands of nanoliter-sized water-in-oil droplets. A droplet with target DNA amplification shows fluorescence and is defined as positive; whereas, a non-fluorescent droplet with no target DNA is defined as negative [15, 16]. The total number of target DNA molecules detected by the ddPCR reaction of a sample is then calculated from the fraction of positive droplets by Poisson statistics [15]. Zhong et al [12] recently reported on a ddPCR assay to detect *C*Las using the 16S rRNA target using plasmid DNA and citrus leaf samples.

The aim of this study was to develop duplex ddPCR assays using primers for 16S rRNA and RNR genes for more accurate, robust, and unambiguous detection of C*Las* in low titer infected citrus leaf and ACP DNA samples. The linearity, dynamic range, sensitivity and low titer detection performance of qPCR and ddPCR duplex assays were compared. The repeatability, reproducibility and tolerance to residual matrix inhibitors for ddPCR assays were performed with *C*Las-infected citrus leaf tissue and ACP extracts.

## Materials and methods

### *C*Las-infected DNA samples

Experimental citrus leaf petiole and ACP samples infected with *C*Las were obtained from the Contained Research Facility, University of California, Davis, California and the U.S. Horticultural Research Laboratory, USDA-ARS, Fort Pierce, Florida. DNA was extracted from citrus leaf and ACP by the cetyl trimethyl ammonium bromide (CTAB) method [17]. Nucleic acid quality and quantity was measured using Qubit 3.0 (Thermo Fisher Scientific, USA).

### Primers and probes

Primers and probes used in qPCR and ddPCR assays are shown in Table 1. The TaqMan probes were synthesized by labeling the 5' -terminal nucleotide with the 6-carboxy-fluorescein (FAM) and VIC reporter dyes for the 16S and RNR genes, respectively. The 3’ -terminal nucleotide was labelled with minor groove binder/non-fluorescent quencher (Thermo Fisher Scientific, USA).

**Table 1:** Primer and probe sequences used for the qPCR and ddPCR assay for detection of “*Candidatus* Liberibacter asiaticus”.

Target Gene	Primer/Probe name	Sequence (5'-3')	Amplicon length	Reference
16S rRNA gene	16S F	TCGAGCGCGTATGCAATACG	76 bp	[11]
	16S R	GCGTTATCCCGTAGAAAAAGGTAG		
	16S P	6FAM/AGACGGGTGAGTAACGCG/ MGB/NFQ		
*nrdB*, β-subunit of ribonucleotide reductase	RNR F	CATGCTCCATGAAGCTACCC	80 bp	[9]
	RNR R	GGAGCATTTAACCCCACGAA		
	RNR P	VIC/CCTCGAAATCGCCTATGCAC/MGB/NFQ		

### Preparation of cloned plasmid and standard curves

The 16S rRNA (76 bp) and RNR (80 bp) genes were amplified using DNA extracted from HLB-symptomatic citrus leaves using 16S F/R and RNR F/R primers, respectively (Table 1). The amplicons were ligated into pGEM-T Easy vector (Promega) and transformed in JM-109 (Promega) separately. Plasmids isolated from white colonies were linearized using SpeI restriction enzyme (New England Biolabs, UK) and the concentrations were measured using Qubit dsDNA BR Assay Kit in Qubit 3.0 fluorometer (Thermofisher). Ten-fold serial dilutions were made using linearized 16S and RNR plasmids to generate the standard curves to assess analytical sensitivity, linearity and dynamic range of the ddPCR and qPCR assays.

*C*Las-infected citrus leaf and ACP DNA were extracted by the CTAB method and used for 10-fold serial dilution ranging from 2 ng to 200 fg per reaction and 1 ng to 100 fg per reaction, respectively. Three replicates of each concentration were tested simultaneously in the same run. The linear relationship was produced by plotting the log DNA concentration against the cycle quantitation (Cq).

### Duplex quantitative qPCR assay

qPCR was performed in a CFX96 Real-Time System (Biorad). Duplex qPCR for ten-fold serial dilutions of linearized 16S and RNR plasmids was optimized using different concentrations of primer:probe (50:100 nm, 100:200 nm, 150:300 nm) in a final volume of 20 μl reaction which contained 10 μl of SsoAdvanced™ Universal Probes Supermix (Biorad), 1 μl of plasmid DNA and final volume made up with double distilled water. The thermocycling conditions consisted of initial denaturation at 95°C for 5 min, then 40 cycles of denaturation at 95°C for 10 s, annealing at 58°C for 40 s. Each run included log10 dilutions of linearized 16S and RNR plasmids, negative control and no template control (NTC).

### Thermal gradient optimization and duplex ddPCR

To determine optimal annealing temperatures for 16S and RNR gene targets, the thermal gradient ranging from 54°C to 64°C was performed in the S1000 thermal cycler (BioRad) using the same amount of linearized plasmid DNA and primers/probes concentrations (900 nM/ 250 nM) in singleplex and duplex assay.

The duplex ddPCR reaction mixture (20 μl) contained 2x ddPCR Supermix for probes (no dUTP) (Biorad), 900 nM of each forward and reverse primer, 250 nM of probe and 1 μl of *C*Las DNA. The reaction mixture was transferred in individual wells of disposable eight channel DG8 cartridge and the wells were filled with 70 μl of droplet generation oil. The prepared cartridge was then placed into a cartridge holder and loaded in to the QX 200 droplet generator. The prepared droplet emulsions were further loaded in a semi-skirted, PCR-clean 96-well plate (Eppendorf) using a multichannel pipet (Mettler-Toledo Rainin LLC, CA, USA), by aspirating 40 μl from the DG8 cartridge. The plate was then heat sealed with pierceable foil using a PX1 PCR plate sealer (Biorad) and PCR amplification was carried out in a S1000 thermal cycler (Bio-Rad). The thermal cycling conditions consisted of 10 min. initial denaturation at 95°C, followed by 40 cycles of denaturation at 94°C for 30 sec. and annealing/elongation at 58°C for 1 min. with a ramp of 2°C/sec. and a final 10 min. incubation at 98°C for enzyme deactivation. After thermal cycling, the plate containing the droplets was placed in a QX 200 droplet reader (Bio-Rad, CA, USA) for analyzing each individual droplet by a detector.

### Precision of *C*Las detection at low titer

To estimate the diagnostic performance of 16S and RNR primers in duplex assay at low target concentrations, *C*Las-positive leaf and ACP DNA (Cq 32) were diluted in healthy citrus DNA extracts at different ratios (1:5; 1:10; 1:15; 1:20; 1:25; 1:30; 1:35 and 1:40). The duplex ddPCR and qPCR assays were performed in ten replicates of each dilution.

### Assessing inter-assay and intra-assay variability of duplex ddPCR

To assess the reproducibility (inter-assay variation) and repeatability (intra-assay variation) of duplex ddPCR assays, triplicate experiments were performed with *C*Las-infected leaf and ACP samples using 16S and RNR primers and probes. The reproducibility was determined by measuring the samples in triplicate within the same experiment (to assess the intra-assay variation) and between three different assays (inter-assay variation). The coefficient of variation (CV) was calculated by standard deviation/mean.

### Estimation of tolerance to inhibitors

The influence of inhibitors with *C*Las citrus leaf and ACP DNA during sample preparation was estimated for the duplex ddPCR assay. The reactions were spiked with the equal amount of *C*Las plasmid DNA (16S and RNR) in different quantities (1 μl to 5 μl) of citrus leaf and ACP extract. The influence of leaf and ACP extracts were assessed relative to the mean measured signals in each sample with no inhibitors (no inhibition control administered by adding double-distilled water to the *C*Las plasmid DNA). To obtain citrus leaf extracts, 0.5 g healthy citrus leaves were excised and homogenized in 10 ml TE buffer (pH 7.4) using a Homex 6 homogenizer (BioReb AG, Switzerland) and centrifuged at 10,000x g for 10 min at 4°C. The healthy ACP was crushed in 0.5 ml of TE buffer and centrifuged at 10,000x g for 10 min at 4°C. The above supernatants were collected and used to test the tolerance of duplex ddPCR assays.

### Data analysis

The standard curves and Cq values for qPCR were generated by Bio-Rad CFX Manager Software version 3.1. Linear regression of the qPCR standard curves were recalculated with Microsoft Excel software (Microsoft, USA). The Cq values were regressed against the logarithmically transformed copy number and DNA concentration. The qPCR amplification efficiency was estimated from the slopes of the standard curves using the equation E = 10^−1/slope^– 1. The ddPCR data were analyzed with QuantaSoft analysis software version 1.7 (Bio-Rad). The positive droplets containing amplified products were discriminated from negative droplets by applying a threshold above the negative droplets. Reactions with more than 10,000 accepted droplets per well were used for analysis. The copy number concentration of each sample was reported automatically by ddPCR software. The linear regression and *P*-value of the ddPCR assay were determined by plotting the measured copies of ddPCR and comparing them with expected values of serial dilution of plasmid DNA, citrus leaf and ACP DNA in Excel. The Poisson error and total error were calculated by QuantaSoft software. To be a true instrument technical replicate, total error bars are always greater than or equal to the Poisson error bars. Receiver operating characteristic (ROC) curves were constructed to evaluate the detection precision of the 16S and RNR primers in duplex ddPCR assay. The t-test was performed to compare the differences in measurement between inhibitors and no inhibitor control by ddPCR assay. Statistical analyses were performed with IBM SPSS Statistics version 24.

## Results

### Singleplex and duplex qPCR assay

**Fig 1:**
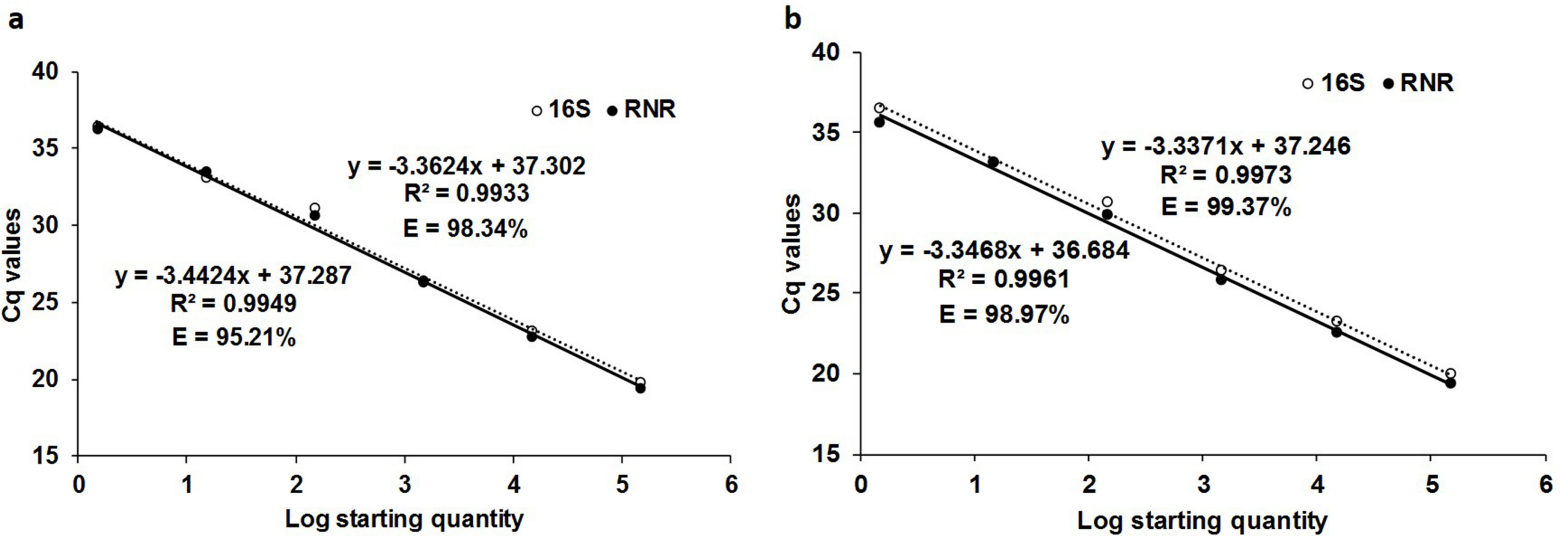
Calibration curve of qPCR singleplex and duplex assays with tenfold serially diluted 16S and RNR plasmid DNA (1.48E+05 to 1.48E+00 copies/μl and 1.47E+05 to 1.47E+00 copies/μl, respectively) using 16S (broken line) and RNR (unbroken line) primers at 100:200 nm primer:probe ratio. (a) The singleplex qPCR assay efficiency for 16S and RNR Plasmid DNA standard curve is 98.34%, 95.21%, respectively, and (b) for duplex qPCR assay is 99.37%, 98.97%, respectively.

The calibration curves at different concentrations of primer:probe (50:100 nm, 100:200, 150:300nm) showed that the duplex qPCR assays had the better linearity and efficiency at a 100:200 ratio with 16S and RNR plasmid DNA (R^2^ = 0.9973 and 0.9961, respectively) over the dynamic range tested in both the plasmid DNA from 1.48E+05 to 1.48E+00 copies/μl and 1.470E+05 to 1.47E+00 copies/μl, respectively. The slopes were -3.3371 and -3.3468, equivalent to qPCR efficiency of 99.37% and 98.97% for 16S and RNR, respectively. The singleplex qPCR efficiencies at 100:200 ratio with 16S and RNR plasmid DNA (R^2^ = 0.9933 and 0.9949, respectively) were 98.34% and 95.21%, respectively (Fig 1A and 1B). The standard curve showed the sensitivities of the duplex qPCR assays were 1.48 copies/20 μl and 1.47 copies/20 μl for both 16S and RNR respectively (S1 and S2 Tables).

The qPCR efficiencies for primer:probe 50:100 nm were 96.82% (R^2^ = 0.9914) and 89.47% (R^2^ = 0.992), for 16S and RNR plasmid DNA, respectively, in singleplex assays and 92.64% (R^2^ = 0.9973) and 92.42% (R^2^ = 0.995), respectively, in duplex assay (S1 Fig). The qPCR efficiency at primer:probe 150:300 nm were 98.12% (R^2^ = 0.9918) and 96.05% (R^2^ = 0.9953), for 16S and RNR plasmid DNA, respectively, in singleplex assay and 94.34% (R^2^ = 0.9839) and 92.42% (R^2^ = 0.9912), respectively, in duplex assay (S2 Fig). Based on these results, a prime:probe ratio of 100:200 nm was further used in this study for quantification of *C*Las in citrus leaf and ACP in duplex qPCR and ddPCR.

**Fig 2:**
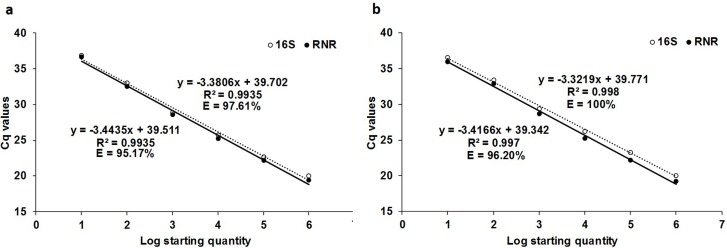
Calibration curve of qPCR singleplex and duplex assays with tenfold serially diluted *C*Las-infected citrus leaf DNA (20 ng to 0.0002 ng) using 16S (unbroken line) and RNR (broken line) primers. (a) The singleplex qPCR assay efficiency for 16S and RNR DNA standard curve is 97.61% and 95.17%, respectively, and (b) for duplex qPCR assay is 100% and 96.20%, respectively.

The *C*Las-infected citrus leaf DNA standard curve showed the efficiencies of 97.61% (R^2^ = 0.9935, slope = -3.3806) and 95.17% (R^2^ = 0.9935, slope = -3.4435) for 16S and RNR, respectively, in singleplex assay (Fig 2A). The duplex qPCR efficiencies were 100% (R^2^ = 0.998, slope = -3.3219) and 96.20% (R^2^ = 0.997, slope = -3.4166), respectively, for 16S and RNR (Fig 2B). The detection limits were 0.0002 ng of leaf DNA for 16S and RNR in duplex assay (S4 and S5 Tables).

**Fig 3:**
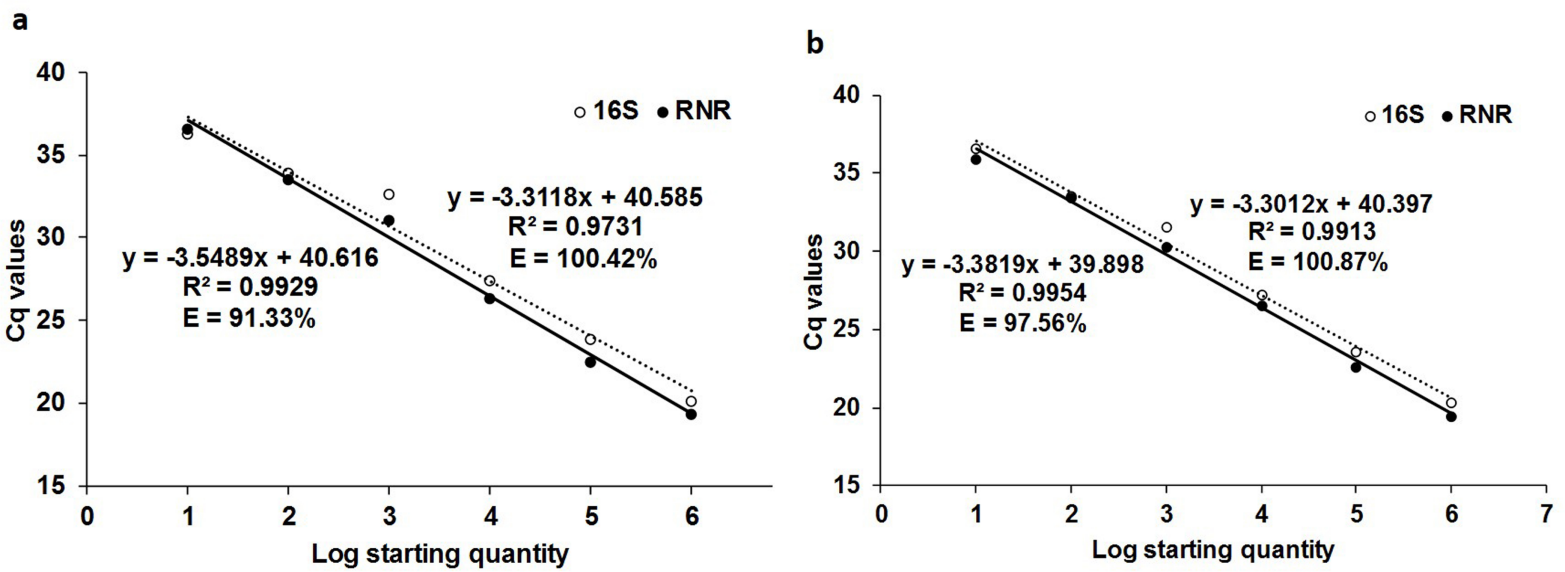
Calibration curve of qPCR singleplex and duplex assays with tenfold serially diluted *C*Las-infected ACP DNA (10 ng to 0.0001 ng) using 16S (unbroken line) and RNR (broken line) primers. (a) The singleplex qPCR assay efficiency for 16S and RNR DNA standard curve is 100.42% and 91.33%, respectively. (b) The duplex qPCR assay is 100.87% and 97.56%, respectively.

The *C*Las-infected ACP DNA standard curve showed the efficiencies of 100.42% (R^2^ = 0.9731, slope = -3.3118) and 91.33% (R^2^ = 0.9929, slope = -3.5489), for 16S and RNR, respectively, in singleplex assay (Fig 3A). The duplex qPCR efficiencies were 100.87% (R2 = 0.9913, slope = -3.3012) and 97.56% (R^2^ = 0.9954, slope = -3.3819), respectively, for 16S and RNR (Fig 3B). The detection limits were 0.0001 ng ACP DNA for 16S and RNR in duplex assay (S6 and S7 Tables).

### Singleplex and duplex ddPCR assay

**Fig 4:**
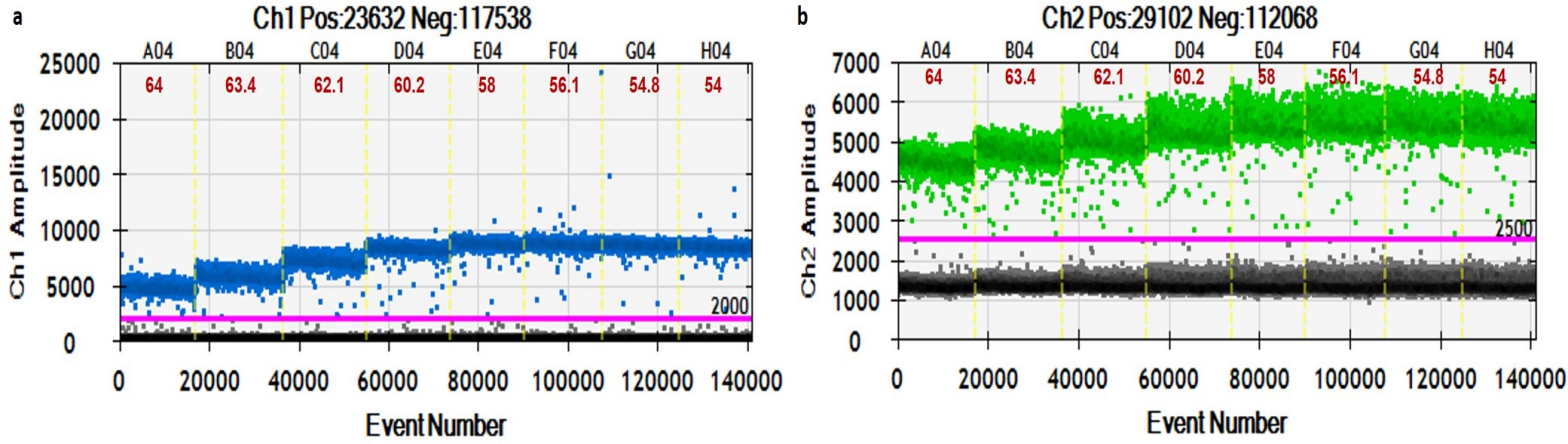
**Thermal gradient duplex ddPCR for optimization of annealing temperature with (a) 16S (b) RNR primers.** Eight ddPCR reactions are divided by vertical dotted yellow lines with an annealing temperature gradient ranged from 64°C to 54°C. The pink line is the threshold, above which are positive droplets (blue and green) and below that are negative droplets (gray) without any target DNA.

The optimum annealing temperature is the one that results in the largest fluorescence amplitude difference between the positives and negatives and that avoids nonspecific amplification. An annealing temperature of 58°C was chosen for the subsequent ddPCR experiments (Fig 4A and 4B).

**Fig 5:**
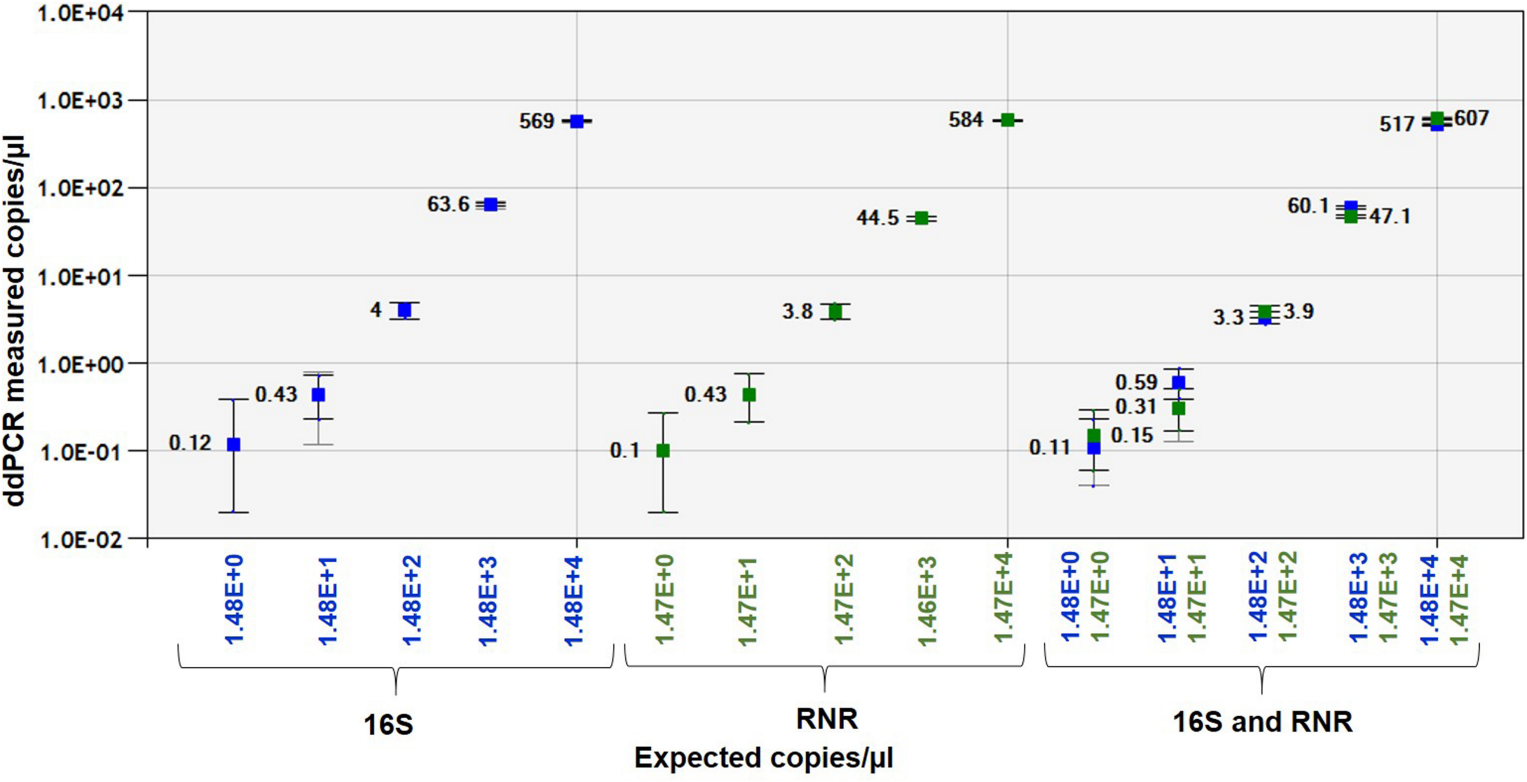
Linear regression of the singleplex and duplex ddPCR assays for Plasmid DNA. The Pearson correlation coefficient of singleplex 16S plasmid DNA regression curve (y = 0.7702x+22.319) is 0.9998 and RNR plasmid DNA (0.7963x-77.668) is 0.9994, respectively. Pearson correlation coefficient of duplex 16S plasmid DNA regression curve (y = 0.6994x+30.482) is 0.9996 and RNR plasmid DNA (0.8275x-76.539) is 0.9995, respectively. The inner error bars indicate the Poisson 95% confidence interval (CI) and the outer error bars show the total 95% CI of replicates. (*P*<0.0001).

The linear regression curve was made by plotting Log10 transformed copy number concentrations measured by ddPCR against Log10-transformed predicted values of serially diluted plasmid DNA in singleplex and duplex ddPCR assays. The 16S and RNR Plasmid DNA showed R^2^ = 0.9998 and 0.9994, respectively, in singleplex assay and R^2^ = 0.9996 and 0.9995, respectively, in duplex assay (Fig 5). The sensitivity of duplex ddPCR assay for 16S and RNR plasmid DNA was 2.2 copy/20μl reaction and 3 copies/20μl, respectively (S3 Table).

**Fig 6:**
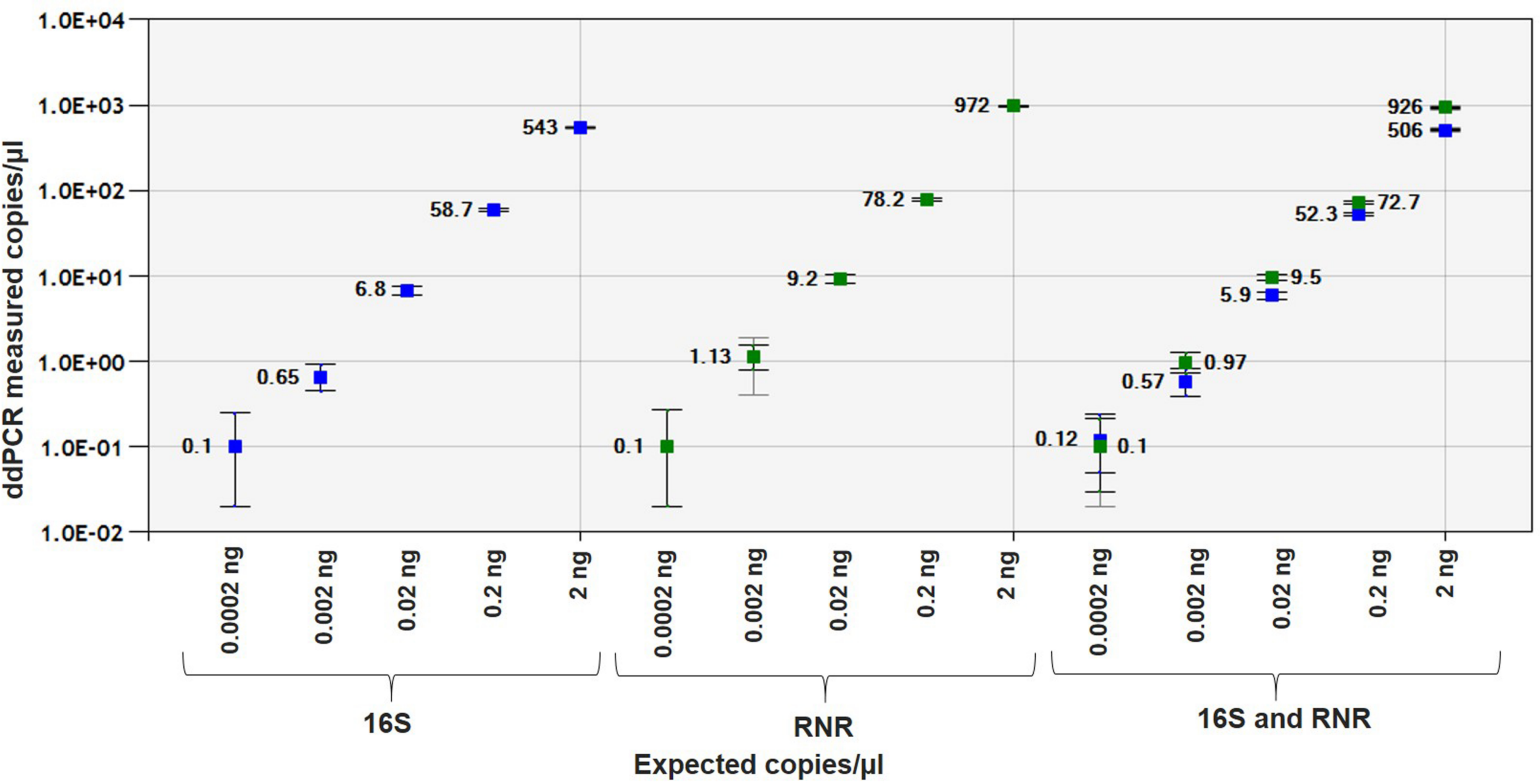
Linear regression of the singleplex and duplex ddPCR assays for *C*Las-infected leaf DNA. Pearson correlation coefficient of singleplex 16S leaf DNA regression curve is 0.9999 and RNR leaf DNA is 0.9996, respectively. Pearson correlation coefficient of duplex 16S leaf DNA regression curve is 0.9999 and RNR leaf DNA is 0.9995, respectively. The inner error bars indicate the Poisson 95% CI and the outer error bars show the total 95% CI of replicates (*P* <0.0001).

The *C*Las-infected citrus leaf DNA showed good linearity for16S and RNR gene with R^2^ = 0.9999 and 0.9996, respectively, in singleplex assay and R^2^ = 0.9999 and 0.9995, respectively, in duplex assay (Fig 6). The sensitivity of ddPCR assay for 16S and RNR was 0.0002 ng (S4 and S5 Tables).

**Fig 7:**
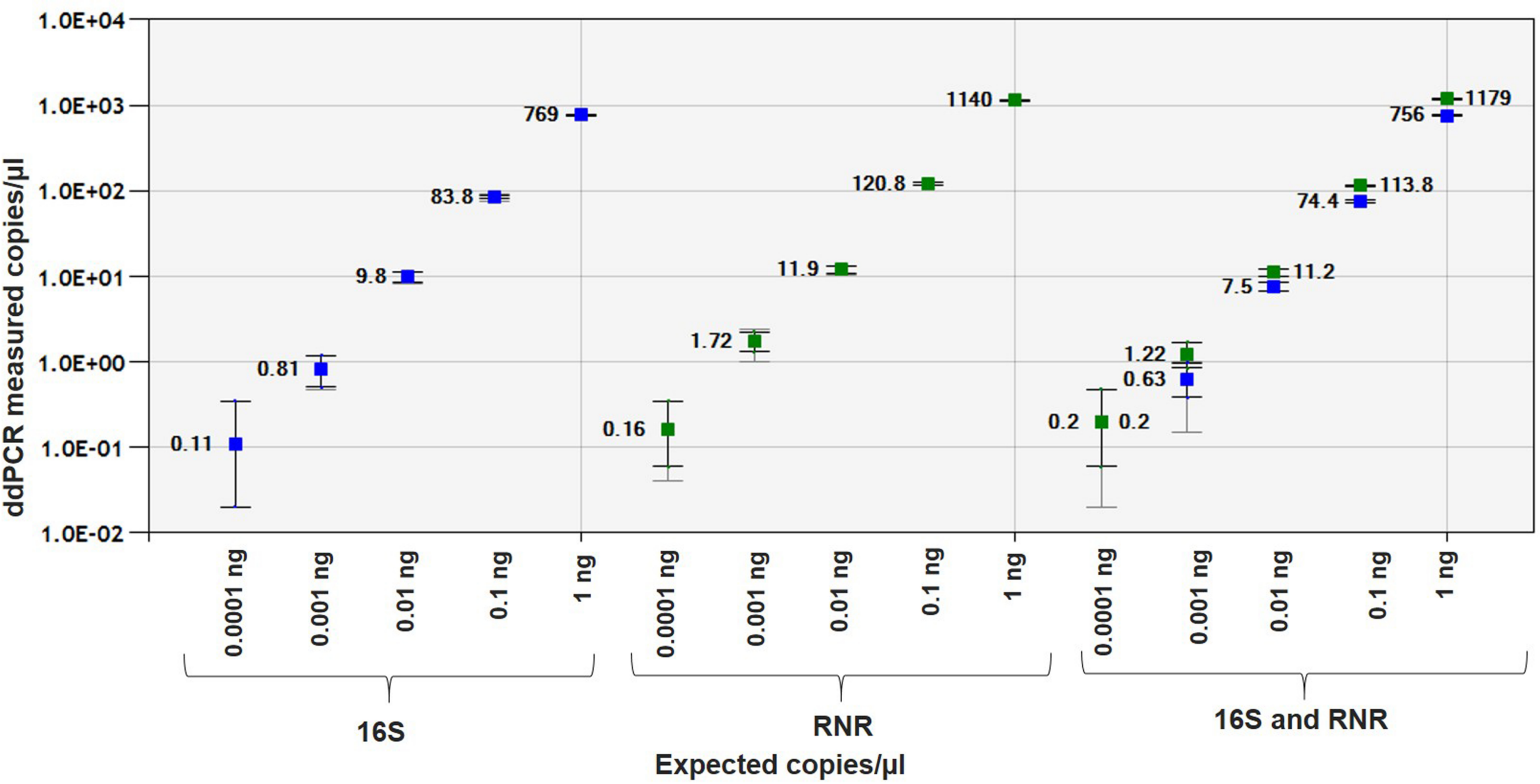
Linear regression of the singleplex and duplex ddPCR assays for ACP DNA. Pearson correlation coefficient of singleplex and duplex assay for 16S and RNR with citrus leaf and ACP DNA regression curve is 0.9999. The inner error bars indicate the Poisson 95% confidence interval (CI) and the outer error bars show the total 95% CI of replicates. (*P*<0.0001).

*C*Las-infected ACP DNA showed good linearity with R^2^ = 0.9999 and 1.0 in singleplex assay for 16S and RNR gene, respectively, and R^2^ = 1.0 for both the genes in duplex assay (Fig 7). The sensitivity of ddPCR assay for 16S and RNR was 0.0001 ng (S6 and S7 Tables).

Singleplex and duplex ddPCR assays showed five orders of magnitude between the target input amounts and ddPCR measured values. Droplets were positively saturated at target concentrations >10^6^ copies/μl, making the Poisson algorithm invalid and resulting in a relative narrower dynamic range compared to qPCR. There were no significant difference (*P*<0.0001) in DNA copy number between singleplex and duplex ddPCR assays with plasmids, citrus leaf and ACP DNA.

### Precision of *C*Las detection at low titer

**Fig 8:**
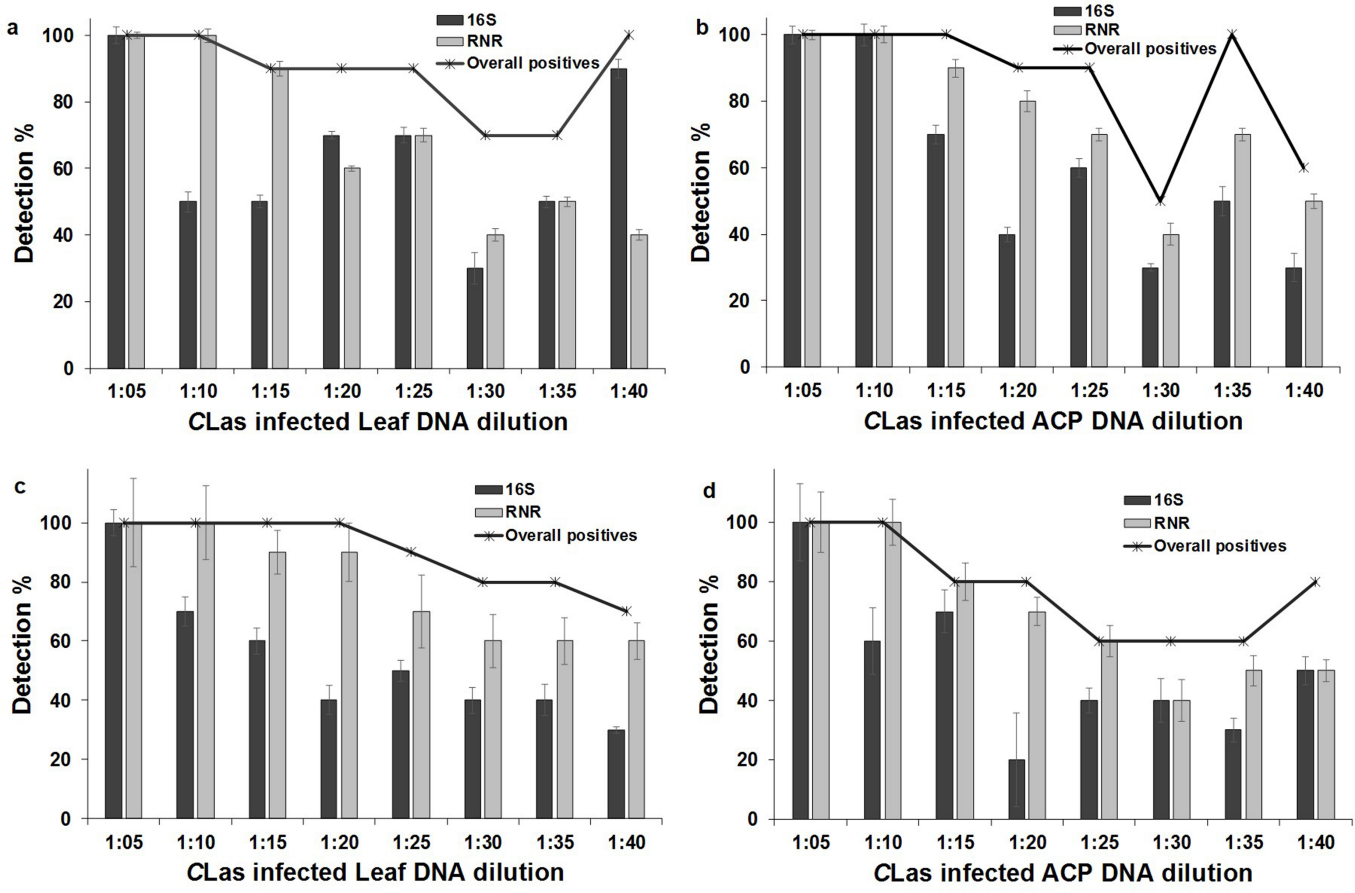
**Precision of detection of *C*Las with citrus leaf and ACP DNA in duplex (a and b) qPCR and (c and d) ddPCR assay.** Equal quantities of *C*Las DNA spiked in healthy extract at different dilution. Error bars indicate standard error of quantification between ten replicates of each dilution. Line shows the overall positive detection rate at different dilutions.

The 16S primers showed 100 percent detection up to a 1:5 ratio, whereas RNR primers showed 100 percent detection up to a 1:10 ratio with leaf and ACP DNA in both qPCR and ddPCR assays. The percent detection of RNR was greater when compared to 16S in all the dilutions with leaf and ACP DNA in both qPCR and ddPCR assays (Fig 8). The duplex ddPCR and qPCR assays showed that the overall positive detection rates were higher with both 16S and RNR primers. The robustness of ddPCR and qPCR assays increased when both 16S and RNR primers were used in duplex assays for detection of *C*Las.

**Fig 9:**
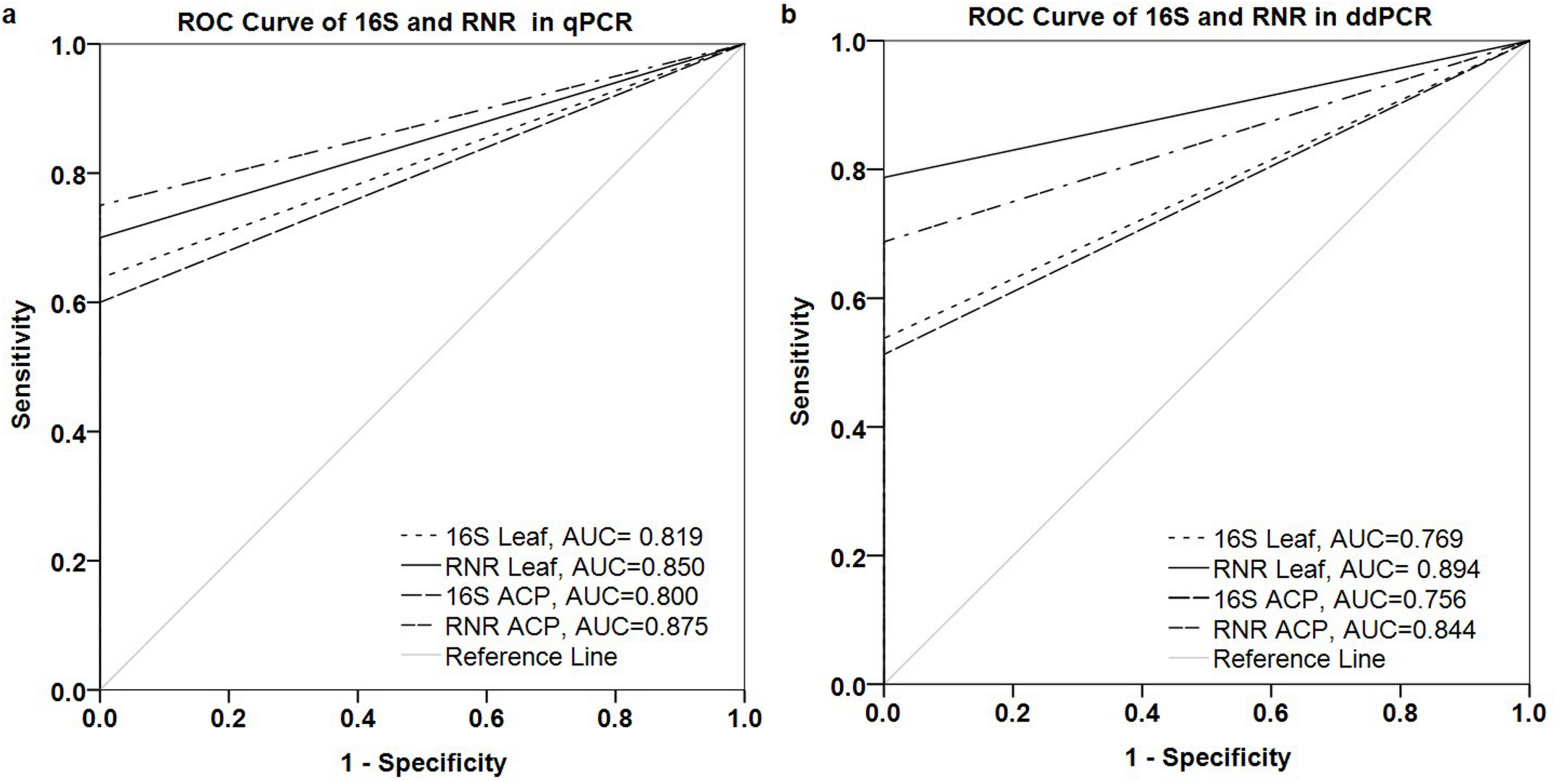
Diagnostic performance of 16S and RNR primers in qPCR and ddPCR assays for *C*Las-infected citrus leaf and ACP DNA. Receiver operating characteristic **(**ROC) curve indicates better diagnostic performance with RNR primer compared to 16S primer for differentiating between healthy and *C*Las infected leaf and ACP DNA with significantly (*P* <0.05) broader AUC in (a) qPCR and (b) ddPCR assays.

In the qPCR assay, the ROC analysis to compare the accuracy of detection between 16S and RNR primers showed that the area under curve (AUC) for 16S was 0.819 (standard error (SE) 0.050, 95% CI 0.720–0.918) and 0.800 (SE 0.054, 95% CI 0.694–0.906) for citrus leaf DNA and ACP, respectively. The AUC for the RNR primer was 0.850 (SE 0.044, 95% CI 0.763–0.937) and 0.875 (SE 0.039, 95% CI 0.798–0.952) for citrus leaf DNA and ACP, respectively (Fig 9A).

In ddPCR assay, ROC analysis between 16S and RNR primers showed that the AUC for 16S was 0.769 (SE 0.060, 95% CI 0.651–0.886) and 0.756 (SE 0.062, 95% CI 0.634–0.878) for citrus leaf DNA and ACP, respectively. The AUC for RNR primer was 0.894 (SE 0.036, 95% CI 0.824–0.963) and 0.844 (SE 0.046, 95% CI 0.754–0.933) for citrus leaf DNA and ACP, respectively. The AUC of RNR primer was significantly (*P <*0.05) broader compared to the 16S primer in qPCR and ddPCR assays, indicating that RNR primer was better than 16S for *C*Las detection in leaf and ACP DNA (Fig 9B).

### Reproducibility and repeatability of duplex ddPCR assay

The absolute quantification of *C*Las for 16S and RNR genes by the ddPCR assay revealed better reproducibility between runs (inter-assay) using RNR primers, especially for low target samples of citrus leaf and ACP. The lower coefficient of variance for 16S primer in intra-assay showed better repeatability within runs compared to RNR primers (Table 2).

**Table 2:** Repeatability (Intra-assay variation) and reproducibility (Inter-assay variation) of duplex ddPCR assay for detection of “*Candidatus* Liberibacter asiaticus".

Assay		Inter-assay variability				Intra-assay variability			
Gene	Leaf/ACP DNA^a^	Assay 1^b^	Assay 2^b^	Assay 3^b^	CV%^c^	Replicate 1^b^	Replicate 2^b^	Replicate 3^b^	CV%^c^
16S	Sample 1	257	283	252	6.3	262	266	257	0.6
	Sample 2	24.4	24.4	25.7	3.0	22.5	22.8	24.4	1.5
	Sample 3	0.9	0.9	1.3	22.3	0.9	1.1	0.9	4.0
	Sample 4	7.3	7.5	6.5	7.5	6.9	8	7.3	2.5
	Sample 5	0.08	0.14	0.09	31.1	0.16	0.9	0.08	16
RNR	Sample 1	405	421	406	2.2	400	416	405	2
	Sample 2	37.2	39.4	37.5	3.1	42.1	39.4	37.2	6.2
	Sample 3	1.8	1.3	2	21.2	1.8	2.3	1.8	14.7
	Sample 4	12.3	11.4	13.6	8.9	12.2	11.4	12.3	4.1
	Sample 5	0.4	0.4	0.36	6.0	0.32	0.15	0.4	44

^a^ Citrus leaf DNA (sample 1–3), Asian citrus psyllid DNA (sample 4, 5)

^b^ Values reflect copies/μl of 20 μl ddPCR reaction

^c^ CV means coefficient of variation

### Influence of inhibitors on duplex ddPCR assay

**Fig 10:**
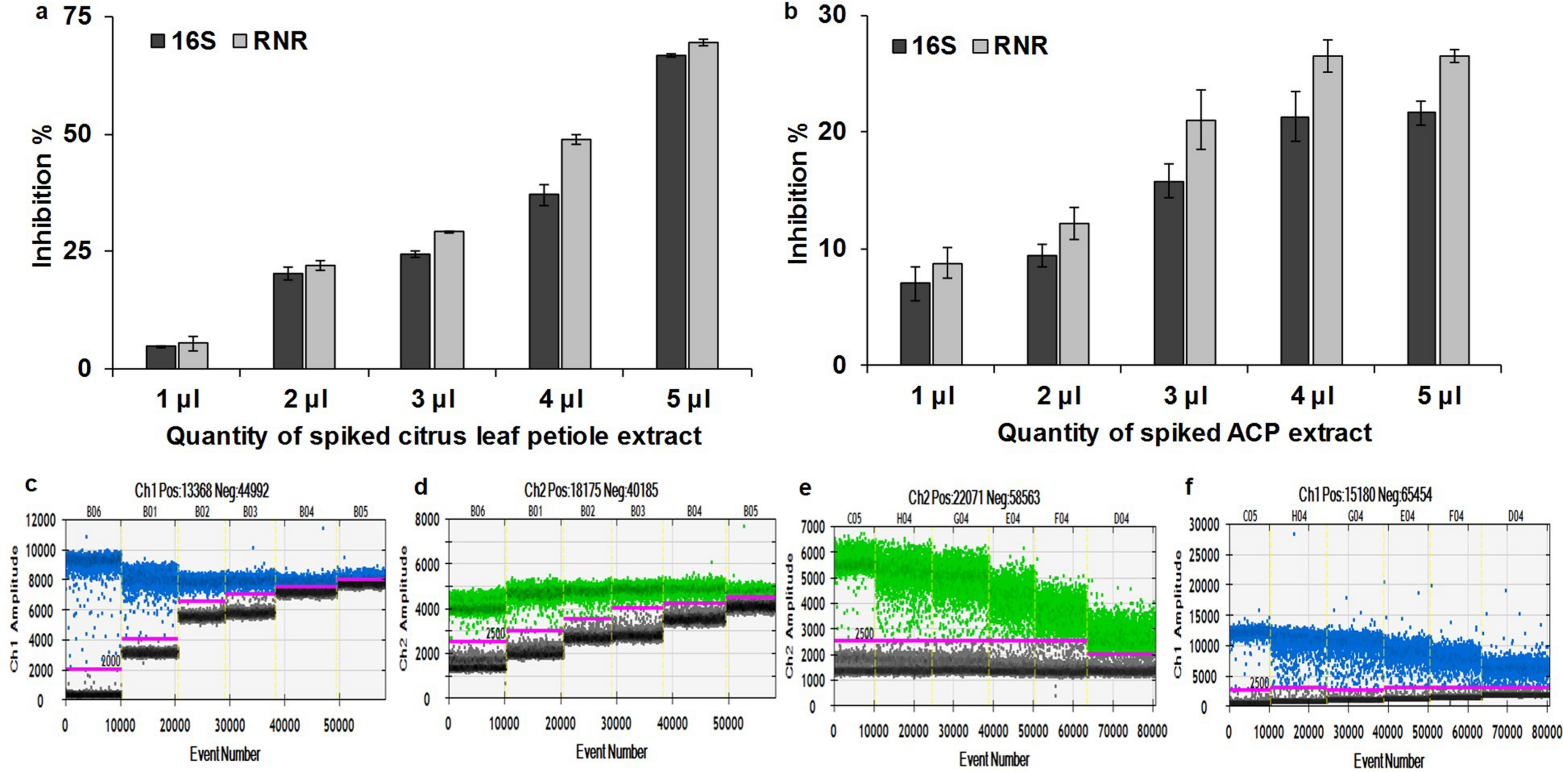
Influence of citrus leaf petiole extract and ACP extract on quantification of *C*Las by ddPCR assays for 16S and RNR genes and 1-D plot of ddPCR reactions. Samples spiked with different quantity of (a) citrus leaf petiole, (b) ACP extract and equal amount of *C*Las plasmid DNA. Error bars denote standard error of inhibition between three replicates of each reaction. The 1-D plot shows only one of three replications for 16S and RNR with citrus leaf petiole (c & d, respectively) and ACP extract (e & f, respectively).

The ddPCR assays showed more tolerance to inhibition with ACP extract compared to citrus leaf petiole extract using 16S and RNR primers (Fig 10A & 10B). In contrast, the key parameters affected by the presence of the residual matrices for the ddPCR were *C*Las titer and fluorescent signal levels for both negative and positive droplets. Fluorescent signals of negative droplets were increased with increasing amounts of spiked citrus leaf petiole extracts and remain similar with ACP extract. Fluorescent signals of positive droplets remained similar and decreased with increasing amounts of citrus leaf petiole and ACP extracts, respectively. The t-test results showed significant (*P* <0.05) difference between different measurements, compare to no inhibition control (Fig 10C, 10D, 10E & 10F).

## Discussion

Currently, the detection of *C*Las is based on 16S rRNA-specific primers for diagnostic and quarantine purposes using citrus leaf and ACP material in the United States [18]. When the pathogen titers are at low levels and unevenly distributed in the infected plant, qPCR based detection of *C*Las is challenging and unreliable due to sampling issues and the presence of inhibitors [19, 20]. The use of a single gene component in qPCR assays for detection of the pathogen with low and variable titers results in high Cq values that are generally considered inconclusive and the pathogen remains unverifiable. The use of two different genomic regions with multi-copy gene characteristics can be beneficial in discriminating between true and false positives [21, 22]. In this study, we developed a duplex assay using primers specific to the 16S rRNA gene (three copies) and the RNR gene (five copies) in qPCR and ddPCR assay that significantly improved the accuracy of *C*Las detection in citrus leaf and ACP.

The absolute quantification of *C*Las using ddPCR, a direct measurement of target titer, compared to qPCR, a relative measurement of titer, showed the ddPCR assay was more precise and reliable confirming results of Zhong et al. [12]. The reliability of titer data generated by qPCR is based on the accuracy of the standard curve obtained from a series of dilutions of known concentrations. This step adds expense, labor, and time for assay completion [23]. The ddPCR has several advantages over qPCR such as absolute quantification without the need of standard curve, improved accuracy, reliability and reproducibility between inter and intra assays [24]. The large-scale partitioning in ddPCR increases the precision of quantification and lessens interference due to PCR inhibitors [25]. The ddPCR technology produces more precise, reproducible and statistically significant results at low concentration of target nucleic acid [26].

In this study, the linearity, dynamic range and sensitivity of ddPCR was compared with qPCR in singleplex and duplex assays. The quantitative detection between the singleplex and duplex assay was not significantly different (*P*<0.0001). Both ddPCR and qPCR assays showed good linearity with plasmid, citrus leaf and ACP DNA. The ddPCR showed saturation of positive droplets at higher concentrations of template, which resulted in lower dynamic range compared to qPCR. The ratio between RNR and 16S copy number was ~1.7 in ddPCR and in qPCR a difference of ~0.7 Cq was observed. RNR primers showed better detection of *C*Las at low titer up to the lowest dilution (1:40) with both infected leaf and ACP. ROC analyses showed that AUC was greater for RNR primers compared to 16S primers for healthy and low titer citrus leaf and ACP samples infected with *C*Las. The higher AUC indicates greater sensitivity and more reliable diagnostic performance. Duplex ddPCR and qPCR assay offered more robust, accurate and sensitive quantification of *C*Las than the singleplex assays based on 16S rRNA or RNR detection. The duplex ddPCR assay exhibited repeatable and reproducible quantitative results with citrus leaf and ACP samples measured at both at high and low *C*Las titer. Therefore, the duplex ddPCR technology utilizing two different multicopy DNA targets in the *C*Las chromosome provided a more robust method for quantitative and unambiguous detection of *C*Las for diagnostic purposes with more precise and reproducible results than qPCR without the need of standard curves.

## Conclusions

To the best of our knowledge, this work is the first to demonstrate absolute quantification of a single pathogen using two multi-copy genes in a duplex ddPCR assay. Our results demonstrated the applicability of simultaneous use of 16S and RNR primers for detecting *C*Las in a duplex ddPCR and qPCR assay. The data on the linearity, dynamic range, repeatability, reproducibility, tolerance to residual matrix inhibitors and the diagnostic performance supports this conclusion. This work suggests that when *C*Las titer is very low, the use of both 16S and RNR targets in duplex ddPCR assay is more reliable than the use of the 16S or RNR target in a singleplex for HLB diagnosis. The duplex assay provided greater reliability, sensitivity and cross reference capability for early pathogen detection in asymptomatic citrus samples.

## Supporting information

S1 Fig Calibration curve of *C*Las qPCR. (a) singleplex and (b) duplex assays with tenfold serially diluted 16S and RNR Plasmid DNA (1.48E+05 to 1.48E+00 copies/μl and 1.47E+05 to 1.47E+00 copies/μl, respectively) using 16S (broken line) and RNR (unbroken line) primers at 50:100 nm primer:probe ratio.
(PDF)

S2 Fig Calibration curve of *C*Las qPCR. (a) singleplex and (b) duplex assays with tenfold serially diluted 16S and RNR Plasmid DNA (1.48E+05 to 1.48E+00 copies/μl and 1.47E+05 to 1.47E+00 copies/μl, respectively) using 16S (broken line) and RNR (unbroken line) primers at 150:300 nm primer:probe ratio.
(PDF)

S1 Table Quantitative data of *C*Las plasmid DNA with 16S primer in qPCR assay. (PDF)

S2 Table Quantitative data of *C*Las plasmid DNA with RNR primer in qPCR assay. (PDF)

S3 Table Quantitative data of *C*Las plasmid DNA with 16S and RNR primers in ddPCR assay. (PDF)

S4 Table Quantitative data of *C*Las infected leaf DNA with 16S primer in qPCR and ddPCR singleplex and duplex assays. (PDF)

S5 Table Quantitative data of *C*Las*-*infected leaf DNA with RNR primer in qPCR and ddPCR singleplex and duplex assays. (PDF)

S6 Table Quantitative data of *C*Las*-*infected insect DNA with 16S primer in qPCR and ddPCR singleplex and duplex assays. (PDF)

S7 Table Quantitative data of *C*Las*-*infected insect DNA with RNR primer in qPCR and ddPCR singleplex and duplex assays. (PDF)
