# Supplementary material for: Development of a duplex droplet digital PCR assay for absolute quantitative detection of "Candidatus Liberibacter asiaticus"
Source: PLoS One. 2018 May 17;13(5):e0197184. doi: 10.1371/journal.pone.0197184 (PMC5957411; doi:10.1371/journal.pone.0197184)
Supplement: S1 Fig — (a) singleplex and (b) duplex assays with tenfold serially diluted 16S and RNR Plasmid DNA (1.48E+05 to 1.48E+00 copies/μl and 1.47E+05 to 1.47E+00 copies/μl, respectively) using 16S (broken line) and RNR (unbroken line) primers at 50:100 nm primer:probe ratio. (PDF) [file pone.0197184.s001.pdf]

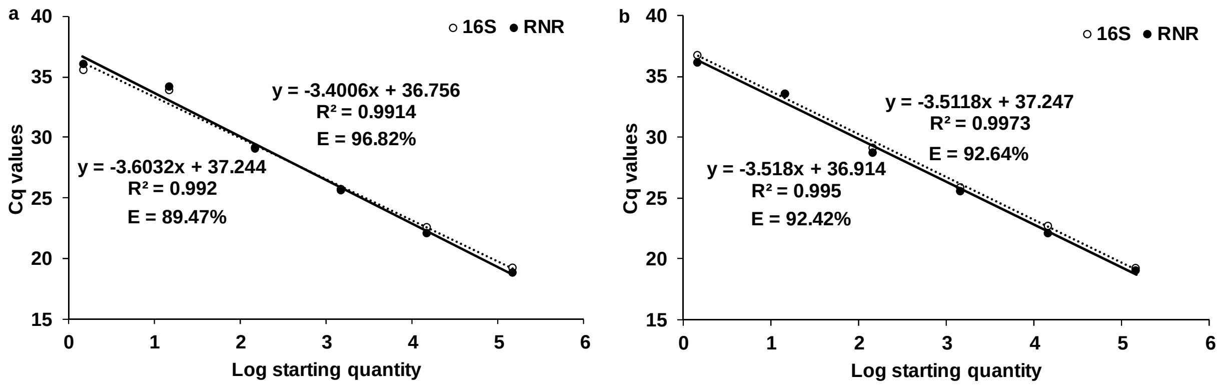

Fig S1. Calibration curve of *C*Las qPCR (a) singleplex and (b) duplex assays with tenfold serially diluted 16S and RNR Plasmid DNA (1.48E+05 to 1.48E+00 copies/µl and 1.47E+05 to 1.47E+00 copies/µl, respectively) using 16S (broken line) and RNR (unbroken line) primers at 50:100 nm primer:probe ratio.
