# Supplementary material for: Development of a duplex droplet digital PCR assay for absolute quantitative detection of "Candidatus Liberibacter asiaticus"
Source: PLoS One. 2018 May 17;13(5):e0197184. doi: 10.1371/journal.pone.0197184 (PMC5957411; doi:10.1371/journal.pone.0197184)
Supplement: S1 Table — (PDF) [file pone.0197184.s003.pdf]

S1 Table. Quantitative data of *C*Las plasmid DNA with 16S primer in qPCR assay

Calculated copies/µl[a]	Log SQ[b]	Primer/Probe concentration											
		50/100				100/200				150/300			
		Singleplex		Duplex		Singleplex		Duplex		Singleplex		Duplex	
		Mean Cq	SD[c]	Mean Cq	SD[c]	Mean Cq	SD[c]	Mean Cq	SD[c]	Mean Cq	SD[c]	Mean Cq	SD[c]
1.48E+05	5.169	19.23	0.090	19.28	0.095	19.81	0.075	20.01	0.111	19.79	0.025	20.16	0.023
1.48E+04	4.169	22.54	0.197	22.74	0.235	23.15	0.146	23.24	0.147	23.11	0.042	23.67	0.044
1.48E+03	3. 169	25.73	0.175	25.86	0.121	26.40	0.112	26.40	0.153	26.42	0.044	26.57	0.471
1.48E+02	2. 169	29.14	0.300	29.09	0.136	31.05	0.526	30.64	0.265	29.88	0.14	30.83	0.276
1.48E+01	1. 169	33.89	0.729	33.57	0.700	33.10	0.485	33.20	0.140	34.33	0.124	35.47	0.375
1.48E+00	0. 169	35.55	0.840	36.72	0.163	36.45	0.352	36.55	0.092	35.94	0.313	36.49	0.417
NTC[d]	0	0	0	0	0	0	0	0	0	0	0	0	0

[a] Values reflect copies/µl of calculated serial dilutions of positive plasmid DNA standard.

[b] SQ means starting quantity. Data represent the mean of each dilution tested in triplicate.

[c] SD means standard deviation.

[d] NTC means no template control.
