# Supplementary material for: Development of a duplex droplet digital PCR assay for absolute quantitative detection of "Candidatus Liberibacter asiaticus"
Source: PLoS One. 2018 May 17;13(5):e0197184. doi: 10.1371/journal.pone.0197184 (PMC5957411; doi:10.1371/journal.pone.0197184)
Supplement: S2 Table — (PDF) [file pone.0197184.s004.pdf]

S2 Table. Quantitative data of *C*Las plasmid DNA with RNR primer in qPCR assay

Calculated copies/µl[a]	Log SQ[b]	Primer/Probe concentration											
		50/100				100/200				150/300			
		Singleplex		Duplex		Singleplex		Duplex		Singleplex		Duplex	
		Mean Cq	SD[c]	Mean Cq	SD[c]	Mean Cq	SD[c]	Mean Cq	SD[c]	Mean Cq	SD[c]	Mean Cq	SD[c]
1.47E+05	5. 168	18.76	0.136	19.03	0.153	19.39	0.123	19.41	0.097	19.32	0.095	19.41	0.021
1.47E+04	4. 168	22.10	0.040	22.12	0.150	22.69	0.358	22.54	0.238	22.51	0.087	22.82	0.104
1.47E+03	3. 168	25.62	0.237	25.56	0.059	26.28	0.097	25.83	0.087	25.83	0.067	25.81	0.469
1.47E+02	2. 168	29.08	0.139	28.72	0.265	30.64	0.295	29.92	0.271	29.34	0.129	30.02	0.265
1.47E+01	1. 168	34.17	0.811	33.57	0.962	33.43	0.292	33.20	0.170	33.70	0.286	34.45	0.256
1.47E+00	0. 168	36.05	0.021	36.16	0.035	36.17	0.395	35.62	0.358	35.84	0.516	36.22	0.679
NTC[d]	0	0	0	0	0	0	0	0	0	0	0	0	0

[a] Values reflect copies/µl of calculated serial dilutions of positive plasmid DNA standard.

[b] SQ means starting quantity. Data represent the mean of each dilution tested in triplicate.

[c] SD means standard deviation.

[d] NTC means no template control.
