# Supplementary material for: Development of a duplex droplet digital PCR assay for absolute quantitative detection of "Candidatus Liberibacter asiaticus"
Source: PLoS One. 2018 May 17;13(5):e0197184. doi: 10.1371/journal.pone.0197184 (PMC5957411; doi:10.1371/journal.pone.0197184)
Supplement: S3 Table — (PDF) [file pone.0197184.s005.pdf]

S3 Table. Quantitative data of *C*Las plasmid DNA with 16S and RNR primers in ddPCR assay

16S							RNR						
**Calculated copies/µl**[a]	**Singleplex**			**Duplex**			**Calculated copies/µl**[a]	**Singleplex**			**Duplex**		
	Mean	Poisson SEM[b]	Total SEM[b]	Mean	Poisson SEM[b]	Total SEM[b]		Mean	Poisson SEM[b]	Total SEM[b]	Mean	Poisson SEM[b]	Total SEM[b]
1.48E+05	NA[c]	NA[c]	NA[c]	NA[c]	NA[c]	NA[c]	1.47E+05	NA[c]	NA[c]	NA[c]	NA[c]	NA[c]	NA[c]
1.48E+04	11380	6.06	14.44	10340	4.33	10.41	1.47E+04	11680	6.06	6.66	12140	4.91	11.27
1.48E+03	1272	1.85	3.77	1202	1.39	1.39	1.47E+03	890	1.59	1.59	942	1.21	1.21
1.48E+02	80	0.46	0.46	66	0.32	0.32	1.47E+02	76	0.46	0.46	78	0.35	0.35
1.48E+01	8.6	0.14	0.20	11.8	0.14	0.14	1.47E+01	5.2	0.12	0.35	6.2	0.10	0.11
1.48E+00	2.4	0.11	0.11	2.2	0.06	0.06	1.47E+00	2	0.07	0.07	3	0.07	0.07
NTC[d]	0	0	0	0	0	0		0	0	0	0	0	0

[a] Values reflect copies/20 µl ddPCR reaction. Data represents the ddPCR values from merged triplicates of each dilutions.

[b] SEM means standard error of mean.

[c] NA means not applicable.

[d] NTC means no template control.
