# Supplementary material for: Development of a duplex droplet digital PCR assay for absolute quantitative detection of "Candidatus Liberibacter asiaticus"
Source: PLoS One. 2018 May 17;13(5):e0197184. doi: 10.1371/journal.pone.0197184 (PMC5957411; doi:10.1371/journal.pone.0197184)
Supplement: S4 Table — (PDF) [file pone.0197184.s006.pdf]

S4 Table. Quantitative data of *C*Las infected leaf DNA with 16S primer in qPCR and ddPCR singleplex and duplex assays

	Singleplex					Duplex				
**DNA concentration[a]**	**qPCR**		**ddPCR**			**qPCR**		**ddPCR**		
	Mean Cq	SD[b]	Mean[c]	Poisson SEM[d]	Total SEM[d]	Mean Cq	SD[b]	Mean[c]	Poisson SEM[d]	Total SEM[d]
20 ng	19.99	0.091	NA[e]	NA[e]	NA[e]	20.05	0.040	NA[e]	NA[e]	NA[e]
2 ng	22.74	0.076	10860	4.33	6.64	23.24	0.035	10120	4.33	17.32
0.2 ng	25.73	0.145	1174	1.30	1.39	26.20	0.020	1046	1.18	1.18
0.02 ng	28.88	0.300	136	0.43	0.43	29.40	0.320	118	0.38	0.38
0.002 ng	33.00	0.105	13	0.13	0.13	33.43	0.590	11.4	0.12	0.12
0.0002 ng	36.87	0.461	2	0.07	0.07	36.55	0.297	2.4	0.06	0.06
Healthy	0	0	0	0	0	0	0	0	0	0

[a] Values reflect concentration of DNA used for serial dilution.

[b] SD means standard deviation.

[c] Values reflect copies/20 µl ddPCR reaction. Data represents the ddPCR values from merged triplicates of each dilutions.

[d] SEM means standard error of mean.

[e] NA means not applicable.
