# Supplementary material for: Development of a duplex droplet digital PCR assay for absolute quantitative detection of "Candidatus Liberibacter asiaticus"
Source: PLoS One. 2018 May 17;13(5):e0197184. doi: 10.1371/journal.pone.0197184 (PMC5957411; doi:10.1371/journal.pone.0197184)
Supplement: S5 Table — (PDF) [file pone.0197184.s007.pdf]

S5 Table. Quantitative data of *C*Las-infected leaf DNA with RNR primer in qPCR and ddPCR singleplex and duplex assays

	Singleplex					Duplex				
**DNA concentration[a]**	**qPCR**		**ddPCR**			**qPCR**		**ddPCR**		
	Mean Cq	SD[b]	Mean[c]	Poisson SEM[d]	Total SEM[d]	Mean Cq	SD[b]	Mean[c]	Poisson SEM[d]	Total SEM[d]
20 ng	19.47	0.090	NA[e]	NA[e]	NA[e]	19.20	0.012	NA[e]	NA[e]	NA[e]
2 ng	22.19	0.106	19440	8.66	15.89	22.19	0.101	18520	6.06	25.12
0.2 ng	25.25	0.081	1564	1.99	2.25	25.30	0.029	1454	1.41	2.05
0.02 ng	28.60	0.142	184	0.64	0.64	28.70	0.165	190	0.49	0.49
0.002 ng	32.54	0.356	22.6	0.21	0.43	32.92	0.320	19.4	0.16	0.16
0.0002 ng	36.69	0.706	2	0.07	0.07	36.00	0.471	2	0.05	0.06
Healthy	0	0	0	0	0	0	0	0	0	0

[a] Values reflect concentration of DNA used for serial dilution.

[b] SD means standard deviation.

[c] Values reflect copies/20 µl ddPCR reaction. Data represents the ddPCR values from merged triplicates of each dilutions.

[d] SEM means standard error of mean.

[e] NA means not applicable.
