# Supplementary material for: Development of a duplex droplet digital PCR assay for absolute quantitative detection of "Candidatus Liberibacter asiaticus"
Source: PLoS One. 2018 May 17;13(5):e0197184. doi: 10.1371/journal.pone.0197184 (PMC5957411; doi:10.1371/journal.pone.0197184)
Supplement: S6 Table — (PDF) [file pone.0197184.s008.pdf]

S6 Table. Quantitative data of *C*Las-infected insect DNA with 16S primer in qPCR and ddPCR singleplex and duplex assays

	Singleplex					Duplex				
**DNA concentration**[a]	**qPCR**		**ddPCR**			**qPCR**		**ddPCR**		
	Mean Cq	SD[b]	Mean[c]	Poisson SEM[d]	Total SEM[d]	Mean Cq	SD[b]	Mean[c]	Poisson SEM[d]	Total SEM[d]
10 ng	20.13	0.044	NA[e]	NA[e]	NA[e]	20.34	0.017	NA[e]	NA[e]	NA[e]
1 ng	23.80	0.155	15380	6.93	6.93	23.65	0.335	15120	6.64	6.64
0.1 ng	27.34	0.160	1676	2.08	4.33	27.26	0.127	1488	1.76	1.76
0.01 ng	32.60	0.359	196	0.69	0.87	31.62	0.071	150	0.55	0.55
0.001 ng	33.87	0.310	16.2	0.20	0.21	33.56	0.341	12.6	0.17	0.24
0.0001 ng	36.22	0.523	2.2	0.10	0.10	36.63	0.070	4	0.12	0.13
Healthy	0	0	0	0	0	0	0	0	0	0

[a] Values reflect concentration of DNA used for serial dilution.

[b] SD means standard deviation.

[c] Values reflect copies/20 µl ddPCR reaction. Data represents the ddPCR values from merged triplicates of each dilutions.

[d] SEM means standard error of mean.

[e] NA means not applicable.
