# Supplementary material for: Development of a duplex droplet digital PCR assay for absolute quantitative detection of "Candidatus Liberibacter asiaticus"
Source: PLoS One. 2018 May 17;13(5):e0197184. doi: 10.1371/journal.pone.0197184 (PMC5957411; doi:10.1371/journal.pone.0197184)
Supplement: S7 Table — (PDF) [file pone.0197184.s009.pdf]

S7 Table. Quantitative data of *C*Las-infected insect DNA with RNR primer in qPCR and ddPCR singleplex and duplex assays

	Singleplex					Duplex				
**DNA concentration**[a]	**qPCR**		**ddPCR**			**qPCR**		**ddPCR**		
	Mean Cq	SD[b]	Mean[c]	Poisson SEM[d]	Total SEM[d]	Mean Cq	SD[b]	Mean[c]	Poisson SEM[d]	Total SEM[d]
10 ng	19.28	0.087	NA[e]	NA[e]	NA[e]	19.50	0.040	NA[e]	NA[e]	NA[e]
1 ng	22.47	0.105	22800	8.95	13.28	22.64	0.342	23580	9.24	9.53
0.1 ng	26.33	0.117	2416	2.42	2.74	26.52	0.116	2276	2.19	2.19
0.01 ng	31.04	0.234	238	0.75	0.81	30.35	0.429	224	0.66	0.66
0.001 ng	33.48	0.320	34.4	0.27	0.40	33.42	0.780	24.4	0.24	0.24
0.0001 ng	36.57	0.200	3.2	0.09	0.09	35.94	0.739	4	0.12	0.13
Healthy	0	0	0	0	0	0	0	0	0	0

[a] Values reflect concentration of DNA used for serial dilution.

[b] SD means standard deviation.

[c] Values reflect copies/20 µl ddPCR reaction. Data represents the ddPCR values from merged triplicates of each dilutions.

[d] SEM means standard error of mean.

[e] NA means not applicable.
